# Supplementary material for: Low-Dose Acetylsalicylic Acid Treatment in Non-Skull-Base Meningiomas: Impact on Tumor Proliferation and Seizure Burden
Source: Cancers (Basel). 2022 Sep 1;14(17):4285. doi: 10.3390/cancers14174285 (PMC9454729; doi:10.3390/cancers14174285)
Supplement: Supplementary file 1 [file cancers-14-04285-s001.zip › cancers-1847160-supplementary.pdf]

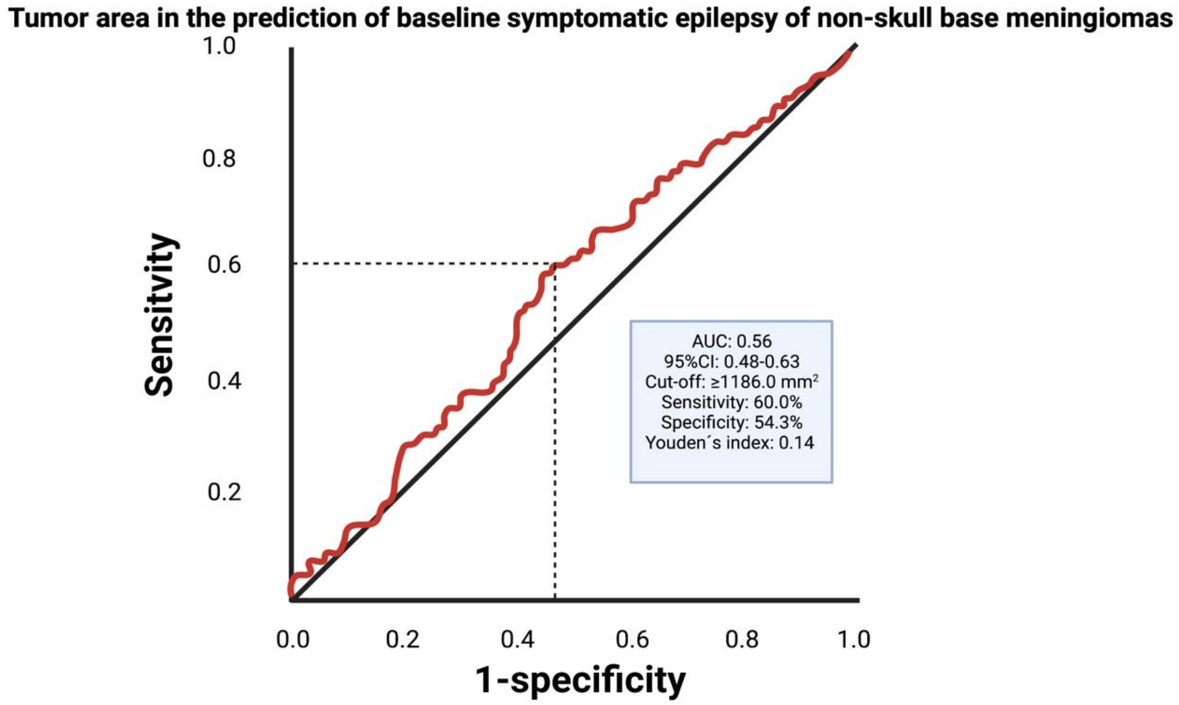

Figure S1. Receiver operating characteristic curve illustrating tumor area in the prediction of preoperative symptomatic epilepsy.

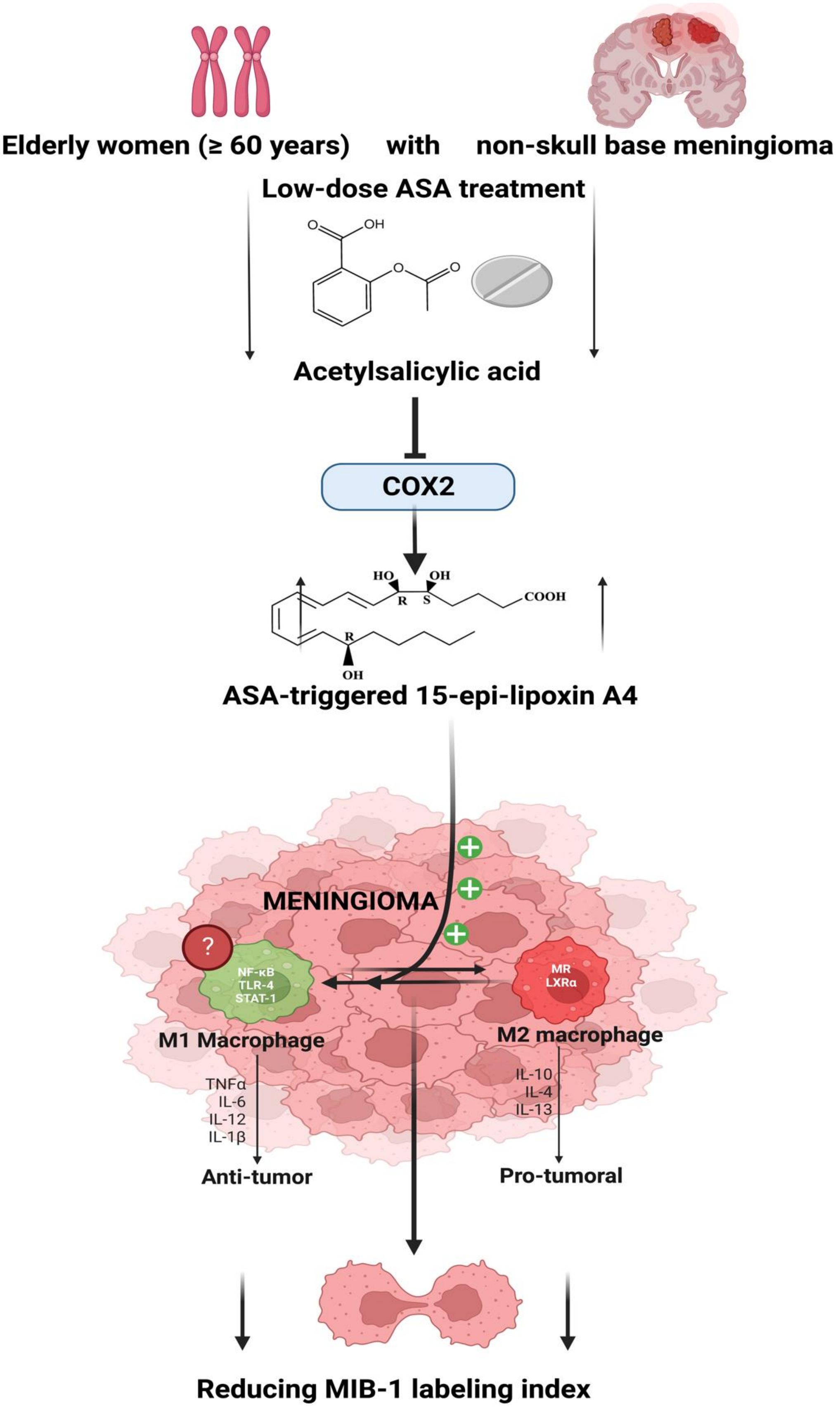

Figure S2. Reduction in MIB-1 labeling index in elderly female non-skull-base meningioma patients by low-dose ASA intake: Hypothesis of a potential pharmacological modulation inducing the formation of M1-phenotype macrophages by ASA-triggered 15-epi-lipoxin A4.
